# A retrospective study of the Hall technique for the treatment of carious primary teeth in Sydney, Australia

**DOI:** 10.1002/cre2.421

**Published:** 2021-04-08

**Authors:** Fani Sapountzis, Tanya Mahony, Amy R. Villarosa, Ajesh George, Albert Yaacoub

**Affiliations:** ^1^ Nepean Blue Mountains Local Health District Oral Health Services Nepean Hospital Kingswood New South Wales Australia; ^2^ Centre for Oral Health Outcomes and Research Translation (COHORT) Western Sydney University Penrith New South Wales Australia; ^3^ Translational Health Research Institute Western Sydney University Penrith New South Wales Australia; ^4^ The University of Sydney Sydney New South Wales Australia

**Keywords:** carious lesions, Hall technique, preformed metallic crowns, primary molars

## Abstract

**Objectives:**

The aim of this retrospective study was to evaluate the outcome of preformed metallic crowns (PMC) utilizing the HT in carious primary molars for children treated within public dental clinics across the Sydney region.

**Materials and Methods:**

A retrospective cohort study was designed, whereby two investigators evaluated 113 primary molars treated with HT PMCs involving 71 participants (aged between 5 and 11 years) after a minimum of 6 months post treatment. The mean time elapsed between crown placement (treatment) and the review was 1.42 years (17 months). The outcome of the HT was assessed by clinical and radiographic criteria.

**Results:**

One hundred thirteen HT PMCs were reviewed from 71 participants. The overall success rate of PMCs placed utilizing the HT was 99%, with only one case presenting with confirmed failure.

**Conclusions:**

HT PMCs have an overall high success rate as a treatment option in carious primary molars.

**Abbreviations**
HT: Hall technique
PMC: preformed metallic crown

## 1 INTRODUCTION

Dental caries is a disease affecting children worldwide (World Health Organization, 2020a). In 2014, the Australian Institute of Health and Welfare (2019) reported an average of 1.5 decayed, missing or filled teeth among children aged 5–10 years, with the prevalence of the decayed component among children aged 10 years ranging from 47% to 63% (Gallagher et al., 2014). Increased consumption of sugar in food and beverages, lack of good oral hygiene practices as well as limited access to dental services (National Advisory Council on Dental Health, 2012) can increase the risk of dental caries. If left untreated in children, dental caries can slow growth, reduce quality of life (Acs et al., 1999) and risk damage to the developing teeth (Welbury, 2017). Current Australian guidelines recommend combined preventive and restorative management of carious primary teeth (NSW Health, 2019).

Traditional approaches to carious lesion management involve complete carious tissue removal and placement of a restoration (Dorri et al., 2017). Clinical decisions are required with respect to the amount of carious tissue removed, the cavity preparation, as well as which material to use to restore the tooth (Maguire et al., 2020).

This treatment may be invasive, often involving destruction of sound tooth structure for access to the carious lesion, particularly in approximal sites (Verde et al., 2009). In addition, conventional restorations have a limited lifetime, leading to a cycle of repeated restorations (Elderton, 1993). Furthermore, traditional approaches may present challenges to clinicians due to lack of patient cooperation, prolonged chair time and/or the need to administer local anesthetic to young children (Threlfall et al., 2005). As a result, the most effective approach for the treatment of the lesion in primary teeth remains the subject of ongoing (Ricketts et al., 2013) and at times vigorous debate (Kidd, 2012).

With greater understandings of the caries process, techniques have been designed to alter the environment of the carious lesion, no longer favoring cariogenic biofilm development, one example of which is the Hall technique (HT) (Ricketts et al., 2013). Developed in Scotland in the 1980's, the HT is a method for managing carious primary molars where carious lesions are sealed under a preformed metallic crown (PMC) without the use of local anesthesia, tooth preparation or any carious tissue removal (Innes & Evans, 2019). The overall aim of the technique is to arrest the carious lesion, with the intention of preserving the tooth until natural exfoliation (Innes & Evans, 2019). The *Hall Technique Guide* (Innes & Evans, 2019) provides criterion for tooth selection, informing clinicians to exclude the HT as a treatment option for teeth with clinical signs of infection, dental abscess, non‐physiological mobility, insufficient hard tissue to retain the crown and radiographic signs of pathology. The *Hall Technique Guid*e states that a tooth with radiographic evidence showing a band of sound dentine, between the lesion and pulp and no indicative evidence of intra‐radicular pathology, is suitable for the placement of a HT PMC. The guide does not state that there must be a minimum thickness of sound dentine prior to HT PMC placement (Threlfall et al., 2005).

The benefits of this treatment are that it may increase patient compliance and operator ease, as well patients' experience of dentistry, as local anesthetic is not required (Innes & Evans, 2019). However, concern remains regarding the influence of the HT PMC on the occlusal vertical dimension (OVD; Van Der Zee & Van Amerongen, 2010). As the placement of HT PMC does not involve any occlusal reduction of the tooth, it is inevitable that placing a crown will result in a premature contact and an increase in the occlusal vertical dimension (Innes et al., 2007). Despite this, Innes et al. (2007) have found that the occlusion can re‐establish within 2–4 weeks, without any symptoms (Gallagher et al., 2014).

Multiple studies have confirmed the overall clinical success of the HT as a treatment modality. In a randomized controlled trial by Innes and Evans (2019), PMCs placed using the HT were found to outperform standard Class II restorations (Innes et al., 2011). Results from the same study showed that only 3% of primary molars treated with the HT experienced major failures (irreversible pulpitis, loss of vitality, abscess) compared with 16.5% of matched control teeth with conventional class II restorations (*p* < 0.001), after a follow‐up period of 60 months (Innes et al., 2011). In addition, a 1‐year follow up study conducted by Santamaria, Innes, Machiulskiene, Evans and Splieth (Santamaria et al., 2014) found the HT PMC to be significantly more successful than both conventional restorations and non‐restorative caries treatment with no major failure after 1 year. In addition, a cost‐effectiveness study conducted in the United Kingdom found the HT PMC to be significantly more economical (*p* < 0.001) when compared to “conventional restorative” approaches (BaniHani et al., 2019).

Although international research has supported the success of the HT in the United Kingdom, Europe, United States of America and New Zealand (Innes et al., 2007; Innes et al., 2011; Ludwig et al., 2014; Santamaria et al., 2014) to date, there have been no similar studies published within a peer reviewed journal in Australia. Since early 2011, the HT has been introduced within public dental clinics in Sydney and has since been utilized as an option in the treatment of carious primary molars by both Dentists and Dental/Oral Health Therapists. Considering the high prevalence of carious lesions among Australian children, it is essential to determine the success of HT when used in public dental settings that manage a large number of pediatric patients (Chrisopoulos et al., 2016). This study is part of a larger quality assurance project, which explored the acceptance of HT PMCs by children and parents/legal guardians, and found the HT PMC to be a well‐accepted restorative treatment option compared to using local anesthetic and/or a conventional restoration, by both children and their parents/legal guardians (Yaacoub et al., 2019).

This study therefore aims to assess the success of HT PMCs on carious primary molars, by retrospectively reviewing routine clinical audit data and patient records at public dental clinics in Sydney.

## 2 MATERIALS AND METHODS

### 2.1 Study design and setting

This retrospective clinical study was conducted using data based on a routine clinical audit across two public dental clinics within the Sydney region. Routine clinical audit data, including records of HT PMC treatments and follow‐up appointments, were retrospectively reviewed as part of this study.

### 2.2 Ethics and anonymity

Ethics approval for this study was obtained from the Human Research Ethics Committee of Nepean Blue Mountains Local Health District (HREC Ref No. LNR/156/NEPEAN/34).

### 2.3 Sampling strategy and eligibility

**FIGURE 1:**
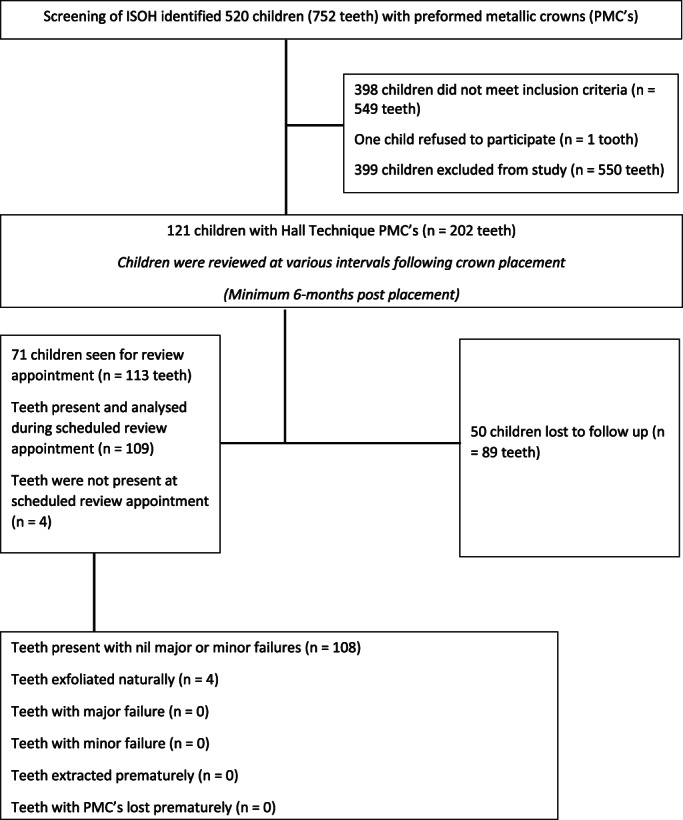
Chart illustrating the flow of participants through the selection criteria

The electronic dental records at the clinical audit sites were reviewed to identify eligible records of children who had received HT PMCs. As seen in Figure 1, patient records were included in the clinical audit if the patient had received a HT PMC. This was determined through a systematic search of the electronic dental records at each site. Using the PMC item number (576), 520 children were identified to have 752 teeth treated with PMCs. Clinical records were then reviewed in detail to ensure participants received a HT PMC. Records were only selected for the study if the patient met the following HT PMC selection criteria (Threlfall et al., 2005):

- Tooth was unrestored prior to placement of the HT PMC.
- Tooth had no tooth preparation in order to fit/cement the HT PMC.
- Used orthodontic separators if approximal contact spaces were closed.
- Tooth had no symptoms of irreversible pulpitis prior to HT PMC placement.
- Tooth had had a clinically diagnostic pre‐operative intra‐oral radiograph taken, revealing a carious lesion that did not extend to the pulp chamber or show inter‐radicular/peri‐apical radiolucency.
- Participant had the HT PMC placed on the tooth for a minimum 6‐month time period.

All HT PMCs reviewed in the clinical audit were cemented using G‐Cem, by GC Corporations, Tokyo Japan.

One hundred twenty‐one children (202 HT PMCs) met the selection criteria for this study and were deemed eligible to participate. Parents/legal guardians of all 121 children were contacted for a scheduled review appointment.

### 2.4 The clinical audit

A routine clinical audit was conducted at the participating study sites among 71 children who received a HT PMC on 113 teeth, treated between January 2011 and January 2015. This treatment was either provided by Dentists or Dental/Oral Health Therapists within the clinic. Parents and/or legal guardians of these children had been contacted to schedule routine review appointments at the clinics, informed of the clinical audit at these appointments and consented for their child to participate in the audit and any required treatment. Data from this clinical audit was recorded on the patient clinical records. Audit data collected included the date of review, dmft/DMFT scores for each child, standard protocol questions relating to history of pain or other symptoms experienced following HT PMC placement and if there was loss of the HT PMC prior to natural tooth exfoliation. Intra‐oral clinical findings such as number of HT PMCs in the mouth and length of time the HT PMC was present were also collected and post‐operative radiographs were taken. This audit was conducted by two independent examiners who reviewed each case and conjointly reached agreement. If there was disagreement, a senior clinician was consulted for consensus. To avoid potential bias, the examiners were also trained and calibrated on clinical and radiographic measures prior to conducting the examination. Follow‐up appointments were then scheduled according to local clinical protocols, for any participants requiring further treatment.

Participants who failed to attend two consecutive scheduled review appointments were also considered as “lost to follow‐up” in the audit.

### 2.5 Data collection

In addition to audit data, dental records and radiographs of the audited cases were reviewed to collect the following data:

- Gender.
- Date of birth.
- Date of HT PMC placement.
- The tooth treated (FDI notation).
- The specific surface with the carious lesion.

The depth of initial carious lesion was based on the examination of the pre‐operative digital radiographic images utilizing the “Caries Management System.” (Evans & Dennison, 2009; Gugnani et al., 2011) Radiographs were standardized for image capture and analysis using a Satelex X‐Mind DC unit (with short cone), 70 kVp,8 mA, film speed “D” used with Scan‐X phosphor plate digital imaging system at an exposure of 0.125 s.

### 2.6 Measures

The primary outcome explored in this clinical audit was status of the HT PMC, which was classified either as a failure or success. A successful treatment indicated that a tooth with the HT had not caused any pain or other symptoms, had no clinical or radiographic pathology present, or had exfoliated naturally without any reported signs or symptoms. The status of the HT was determined according to the outcomes of four assessments: (i) clinical examination; (ii) current pain or pain history prior to natural tooth exfoliation and/or extraction; (iii) percussion and palpation testing; (iv) radiographic assessment. The secondary outcome was degree of failure, which was either classified as a minor failure or major failure, as previously classified by Innes et al. (2006) to facilitate comparison (Innes et al., 2006, 2007). A minor failure constituted HT PMC failure which could be managed by repair or replacement of the HT PMC, whereas a major failure involved signs and symptoms of irreversible pulpal damage, such as dental abscess, broken or unrestorable tooth (Table 1).

**TABLE 1:** Outcome criteria for the clinical and radiographic assessment of HT PMC treated primary molars

Success 112 teeth, 70 children	Major failure 1 tooth*, 1 child	Minor failure 0 teeth, 0 children
Treatment satisfactory with no further intervention required	Presence of dental abscess	Presence of new carious lesion (around HT PMC margins)
No symptoms	Signs/symptoms of irreversible pulpitis	Perforation of the crown
No radiographic/clinical pathology present	Presence of inter‐radicular or peri‐apical radiolucency on radiographs*	Impaction of the HT PMC on the eruption of the first permanent molars
Tooth naturally exfoliated with no “major” or “minor” failures	Presence of internal resorption on radiographs*	Crown loss prior to tooth naturally exfoliating

The outcome of the tooth treated by the HT was then categorized as either a success, minor failure or major failure, based on both the pre‐operative and post‐operative clinical and radiographic findings, using the criteria shown in Table 1.

### 2.7 Statistical analysis

Data were entered into IBM SPSS for Windows, version 25 (SPSS Inc., 2017), where all analysis was undertaken. Descriptive statistics were computed to represent the number, location, depth of carious lesion and involved surfaces of molars treated with the HT, as well as the HT PMC status at the time of review.

## 3 RESULTS

From the original data collection, 520 children (752 teeth with PMCs) were identified as having been provided the item number for PMCs. Out of these, 121 children (202 HT PMCs) met the selection criteria for this study and were deemed eligible for inclusion. Fifty participants (89 HT PMCs—41.3%) were not followed up as part of the clinical audit, thus could not be included in this study. A total of 113 HT PMCs (71 participants—58.7% of eligible patients) were included in analysis, with no missing data for any participants or PMC's in this study. A total of 66 (58.4%) HT PMC's were provided to females and 47 (41.6%) to males. At the time of HT PMC placement, the average age of participants was 6.1 years, with the mean age at follow‐up being 7.5 years (range = 5–11 years). On average, 16.8 ± 6.1 months elapsed between HT PMC placement and review, where the success or failure of the treatment was determined. There was no significant difference between males and females on the age at placement, age at review, or time elapsed between placement and review. See Table 2 for full demographic characteristics of the study sample.

**TABLE 2:** Demographic characteristics of children at the provision of each HT PMC

Sex	N (%)
Male	47 (42)
Female	66 (58)
Age in years at HT PMC placement (mean ± *SD*)^a^	6.1 ± 1.4
Age in years at review appointment (mean ± *SD*)^a^	7.5 ± 1.4
Duration in years since HT PMC placement (mean ± *SD*)^b^	1.43 ± 0.49
dfmt/DMFT score at review appointment (mean ± *SD*)^b^	5.32 ± 2.975

^a^ Total N participants = 71.

^b^ Total N PMCs = 113.

Among study participants, there was a median number of one HT PMC placed per child. This was also the mode number of HT PMC's placed, with almost 60% (*n* = 42) of participants having received a single HT PMC, and less than 10% (*n* = 6) having received more than three HT PMCs (Table 3).

**TABLE 3:** Number of HT PMCs per child

Number of HT PMCs^a^	N (%)
1	42 (59)
2	21 (30)
3	4 (6)
4	2 (3)
5	1 (1)
6	1 (1)

^a^ Total N PMCs = 113.

**FIGURE 2:**
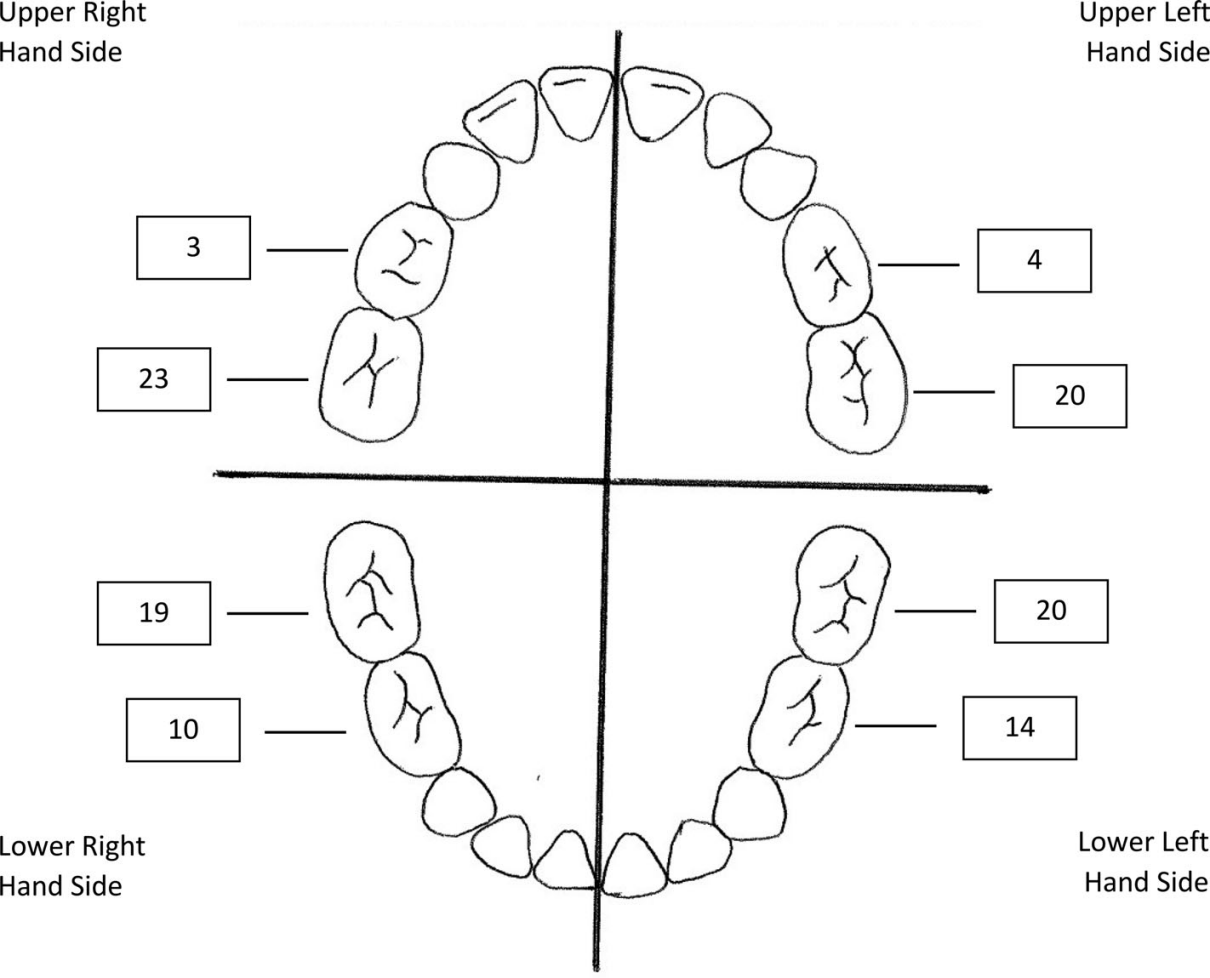
Distribution of primary molars treated using HT PMCs

At the review appointments, 109 teeth (96.5%) of the total 113 teeth, which received HT PMCs were still present and four (3.5%) had naturally exfoliated. As shown in Figure 2, 50 maxillary teeth and 63 mandibular teeth were treated with HT PMCs including 31 first primary molars and 82 second primary molars. There were 55 molars treated in the right quadrants, compared to 58 in the left quadrants. Overall, the maxillary right second primary molar was the most commonly treated tooth (19.3%) using HT PMCs. The approximal surfaces were the most common tooth surface presenting with a carious lesion prior to HT PMC placement (50.4%), followed by occlusal surfaces (37.2%). Radiographically, over two thirds of participants had carious lesions limited to the outer third of dentine (67.3%). Refer to Table 4 for all HT characteristics.

**TABLE 4:** Characteristics of placed HT PMCs

	N (%)
Radiographic depth of lesion^a^	
Enamel only	1 (0.9)
Up to outer third of dentine	79 (67.3)
Inner two thirds of dentine	23 (20.4)
Inconclusive	13 (11.5)
Tooth surface presenting with carious lesions	
Occlusal only	42 (37.2)
Approximal only	57 (50.4)
Both occlusal and approximal	14 (12.4)

^a^ Total N PMCs = 113.

Of a total of 113 teeth that we included in the study, 112 (99.1%) met the success criteria. These teeth were classified as a success, as they did not require any further intervention following treatment, were asymptomatic, did not have any radiographic/clinical pathology present and/or exfoliated without any clinical complications. Only one tooth still present at the time of review was identified to have a major failure, and none with minor failures. The major failure was attributed to the presence of inter‐radicular and peri‐apical radiolucency on the review radiograph. The tooth was identified within Group 3 of carious lesions on the occlusal surface, with caries extending over two thirds into dentine, but not involving pulp, as seen on the pre‐operative radiographs. The female child who presented with the HT PMC failure was aged 4.9 years at the time of placement, and had a HT PMC placed on the maxillary right second primary molar. The failure was identified 1.86 years after placement and required the tooth to be extracted. Due to only a single failure, the association between other variables and failure as well adjustment for multiple measures could not be further explored.

## 4 DISCUSSION

Since the introduction of the HT in the late 1980's as a different approach to conventional PMCs, there have been numerous published studies documenting the effectiveness of a biological approach to the management of the carious lesion (Ludwig et al., 2014). However, there is limited published research in Australia that demonstrates the success of the HT among children.

In the current study sample, the mean age of children who received a HT PMC was 6.1 ± 1.4 years‐of‐age, 41% of participants received multiple HT PMCs. This is consistent with other international studies, where the mean age of children who received a HT PMC was between 5.1 and 6.8 years (Clark et al., 2017; Innes et al., 2006, 2007; Ludwig et al., 2014). In this present study, the mean review time after placement was 1.4 ± 0.5 years (17 months), while in two other studies with similar designs, the mean observation time ranged from 9.9 ± 6.5 months (Clark et al., 2017) to 15 months (Ludwig et al., 2014). Both studies however had a similar high success rate to the current study (97% and 98.9%, respectively) (Clark et al., 2017; Ludwig et al., 2014). Innes et al. (2006) reported that the mean number of HT PMCs fitted per child was four, however in the current study it was 1.6 HT PMCs. The dmft/DMFT at the time of review has been calculated in previous studies as 6.04 ± 2.97 (Santamaria et al., 2014). In comparison, the present study calculated the dmft/DMFT as 5.32 ± 2.97.

The most commonly treated tooth in this study was the maxillary second primary molar, with the most common carious lesion site being the approximal tooth surface. Further, the majority of HT PMCs were placed for carious lesions identified radiographically within the outer third of dentine. Radiographs assist clinicians in the detection of carious lesions at an earlier stage than by visualization alone, particularly for approximal lesions and for occlusal lesions in dentine (Gomez, 2015; Kidd & Pitts, 1990). Innes et al. (2007) reported HT PMC placements to be most common on mandibular first primary molars (32%), with the most common area of caries being the approximal surface (68%) and with 58% of lesions extending less than halfway into dentine (Innes et al., 2007). There are mixed views around the association between the depth of carious lesions and HT success or failure with one study showing no association (Boyd et al., 2018) while another finding the failure rates increase when the carious lesion is more than halfway into dentine (Innes et al., 2007). Interestingly, Boyd et al. (2018) concluded that when baseline carious lesions had marginal ridge breakdown, there was a higher proportion of successful outcomes in the HT group, when compared to conventional restorations.

The results of the present study highlighted that the HT was highly successful when used to treat carious lesions in primary molars, identifying one failure. However, researchers were unable though to determine any associated factors with HT PMC failure due to this low failure rate. These results are consistent with international studies including a Scottish study which utilized a split mouth design comparing HT PMCs to conventional restorations, showing a statistically significant success rate at 23 months. There were three major failures (2.41%) due to symptoms of irreversible pulpitis and dental abscess and six minor failures (4.84%) due to HT PMC loss, HT PMC worn through and secondary caries detected around the margins (Kidd, 2012). The results from a 5‐year randomized controlled trial by Innes, Evans and Stirrups, showed PMCs placed using the HT outperformed standard Class II restorations and illustrate likely long‐term effectiveness of HT PMC's (Innes et al., 2011). Results from the same study showed that teeth treated with the HT had significantly fewer major failures than matched control teeth with conventional class II restorations (3% vs. 16.5%, *p* < 0.001) (Innes et al., 2011). A similar study, completed in the United States, also demonstrated a 97% success rate (Ludwig et al., 2014) with two failures of HT PMCs determined by abscess formation. Clark and colleagues, reported that ectopic eruption of the first permanent molars was also an indicator of failure (Clark et al., 2017).

The survival rate seen in the present study after a mean review time of 17 months, is also consistent with previous findings in exceeding the success of all other methods used to restore primary molars at 1‐ and 2‐year follow‐up (Hickel et al., 2005; Ludwig et al., 2014). In contrast though, one study found that the risk of HT PMC failure was highest during the first 2 years following placement (Ludwig et al., 2014). Nevertheless, the high success of the HT can be attributed to its non‐invasive design, durability and the use of biological biofilm alteration at individual tooth level to arrest the carious lesion (Kidd et al., 2008; Santamaria et al., 2014). It is known that carious imbalances can be eliminated by changes to the oral environment by removal of biofilm, increasing saliva, reducing sugar frequency or by physically blocking the cariogenic biofilm from its substrate (Dawes, 1996; Dirks, 1966; Hara & Zero, 2010). The HT PMC manipulates the plaque environment by sealing it into the tooth, separating it from the substrate that it would normally receive from the oral environment (Innes & Evans, 2019).

The present study has several limitations. Firstly, as the study was conducted retrospectively, it was difficult to ensure that there was no bias from parents, children and/or dental professionals in the decision to use the HT as a treatment option. This also prevented the researchers from comparing the HT PMCs against other treatment modalities. Secondly, this study was undertaken in only one region of Sydney, Australia, which could potentially affect the generalizability of results. This study had a shorter time between treatment and follow‐up compared to other studies, on average 1.4 years compared to more than 2 years (Innes et al., 2007), which may reduce the number of failures seen. Although all clinicians followed a standard protocol when providing and recording treatment, data was collected retrospectively and thus variation in the initial recorded information may exist, which may impact the validity or reliability of study data. In addition, 41.3% of participants were lost to follow‐up, which may have also introduced bias, with studies identifying that those who are less likely to present to the dental clinic may be more likely to experience treatment failure (Ludwig et al., 2014). These participants may not have returned for a number of reasons, including but not limited to: moving out of area, inability to contact, lack of attendance, negative dental experiences, accessing care with an alternative provider or if the tooth became symptomatic prior to the scheduled review appointment. Researchers in the current study were unable to collect the demographic data or dmft/DMFT scores for patients lost to follow‐up, therefore were unable to determine any differences in characteristics, or impacts on success rate. Additionally dmft/DMFT scores collected at the review appointment represented only “unmistakable” cavitated lesions, which may have led to an under representation of the true prevalence or experience of dental decay (World Health Organization, 2020b).

Although no participants were excluded on the basis of age, no participants younger than 5 years old or older than 11 years old participated in the study, as these age groups often did not meet the selection criteria. This may impact the applicability of findings to children of other ages. Finally, like Ludwig et al. (2014) the present study did not achieve a statistically significant comparison owing to the infrequent incidence of failure relative to sample size, which resulted in low statistical power. An increase in sample size and prospective study design would allow for further definitive correlations between failures and associations. Despite the limitations of this study, this is the first published study of its kind in Australia, and will provide valuable insight to this area, which can be explored in future studies.

Overall, these results support the concept of caries control managing the activity of the biofilm. HT PMCs allow an effective, durable seal for a multi surface cavity, it is a predictable restorative option with low failure and re‐treatment rates for managing carious primary molars in a primary health care environment (Innes et al., 2011).

## 5 CONCLUSION

The results of this study support the clinical success of the HT and provides valuable insight into the use of HT PMCs as a viable restorative technique in Australia, having relevance to dental professionals, parents and their children. There is a need for further research into the longer‐term success of the HT, including its success across a wider age range, effectiveness based on varying depths of carious lesions, cost effectiveness in the Australian context, clinicians' preference and use of the HT, as well as comparison to other current restorative methods.

## CONFLICT OF INTEREST

The authors declare no potential conflicts of interest of this article. The ICMJE Form for Disclosure of Potential Conflicts of Interest has been completed by all authors.

## AUTHOR CONTRIBUTIONS

Fani Sapountzis, Tanya Mahony and Albert Yaacoub conceptualized the study and assisted in the data collection. Amy R. Villarosa and Ajesh George were involved in the data analysis. All authors contributed to the preparation and critical revision of the manuscript, and agree to be accountable for all aspects of the study.

## FUNDING INFORMATION

None.

## Data Availability

The data that support the findings of this study are available from the corresponding author upon reasonable request.
